# Hybrid Tandem White Light-Emitting Diodes Based on GaN and Organic Emitters

**DOI:** 10.3390/ma18245684

**Published:** 2025-12-18

**Authors:** Jin-Zhe Xu, Xiao-Zhao Zhu, Feng Zhai, Wei-Zhi Liu, Dong-Ying Zhou, Liang-Sheng Liao

**Affiliations:** 1 Institute of Functional Nano & Soft Materials (FUNSOM), Jiangsu Key Laboratory for Carbon-Based Functional Materials & Devices, Soochow University, Suzhou 215123, China; 20244214144@stu.suda.edu.cn (J.-Z.X.); 20224014009@stu.suda.edu.cn (W.-Z.L.); 2 Institute of Organic Optoelectronics (IOO), Jiangsu Industrial Technology Research Institute (JITRI), Suzhou 215215, China; zhuxiaozhao@metasight.com.cn; 3 Key Laboratory of Optoelectronic Technology and Systems (Ministry of Education), College of Optoelectronic Engineering, Chongqing University, Chongqing 400044, China; zhaifeng@yeechips.com; 4 Macao Institute of Materials Science and Engineering, Macau University of Science and Technology, Taipa 999078, Macau SAR, China

**Keywords:** high-resolution microdisplays, tandem, white LEDs, GaN, OLED

## Abstract

Tandem white organic light-emitting diodes (OLEDs), formed by stacking red, green, and blue organic electroluminescent units, offer a promising route toward high-resolution microdisplays. However, their performance is constrained by the intrinsically short lifetime of blue OLED sub-units. Replacing the unstable blue OLED with a long-lived GaN-based LED could address this limitation, but practical hybridization remains difficult because of incompatible fabrication routes and significant current imbalance between the inorganic and organic units. Here, we demonstrate the first hybrid GaN–OLED tandem white LEDs enabled by an interface-engineered charge-generation unit (CGU). By introducing an ITO/HAT-CN/LiNH_2_-doped Bphen CGU, we simultaneously enhance the work function, strengthen the built-in electric field, and smooth the interfacial morphology. These synergistic effects promote efficient charge generation, yielding near-ideal voltage summation and well-balanced electron–hole injection. As a result, the hybrid tandem device shows a nearly twofold increase in current efficiency (from 28.1 to 58.6 cd A^–1^) and significantly reduced spectral shift under varying current densities. We further demonstrate the generality of this approach by integrating the GaN emission with yellow OLEDs to produce stable blue–yellow hybrid white emission. This work establishes an applicable strategy for integrating GaN-LEDs and OLEDs, opening a pathway toward efficient, stable, and compact white light engines for next-generation microdisplay technologies.

## 1. Introduction

High-resolution microdisplays are becoming essential components in emerging near-eye systems such as virtual and augmented reality [1,2,3]. Achieving pixel densities exceeding 2000 ppi is particularly critical for realizing compact optical engines with high visual fidelity. Current microdisplay technologies include liquid crystal on silicon (LCOS) [4,5], organic light-emitting diode (OLED) [6,7], and micro-LED [8,9]. Organic materials often exhibit some remarkable properties when exposed to voltage or laser irradiation, such as random carbon nanotube networks [10] and phosphorescent materials, etc. OLED, leveraging the unique advantages of organic materials, has attracted the attention of many researchers in the fields of solid-state lighting and display. OLED and micro-LED have gained broader interest due to their higher contrast, faster response times, and lower power consumption, making them more promising candidates for next-generation microdisplay applications.

Despite their advantages, OLED- and micro-LED-based microdisplays still face critical challenges when pixel sizes shrink to the sub-10 µm regime required for ultra-high resolutions. For OLEDs, pixel definition relies on fine metal masks (FMMs), whose mechanical limitations and shadowing effects restrict the minimum achievable pitch and lead to significant fabrication non-uniformity. To solve this problem, the researchers explored many different approaches. Kim et al. have developed a method of applying vacuum-deposited fluorine-containing photoresist and performing lithography, which has enabled the OLED pixels [11]. Another approach is to improve the quality of the mask. There have been reports of preparing ultra-high-definition (UHD) FMM [12]. However, the cumbersome procedures of lithography have also hindered its further development, and high-quality FMM increased manufacturing costs. Micro-LEDs, which are intrinsically bright and efficient, can be integrated with field-effect transistors (FET), to achieve excellent microdisplays. Lee et al. have studied the related electrical properties after integrating with carbon nanotube FETs, and proved their excellent electro-optical conversion characteristics [13]. However, white micro-LEDs depend on the color conversion or massive transfer, like the transfer-printed method of individual RGB chips [14], making it difficult to assemble high-pixel-density displays with sufficient accuracy. Some researchers are dedicated to exploring green and even red GaN-LEDs, but the preparation of these two types of LEDs is very challenging, and their efficiency is also lower compared to that of blue GaN-LEDs [15].

A practical route currently adopted in microdisplays is the use of white OLEDs combined with color filters on a complementary metal–oxide–semiconductor (CMOS) backplane [16]. This approach circumvents the resolution limits imposed by FMMs and the precision-assembly requirements of RGB micro-LED arrays, allowing more reliable scaling to ultra-high pixel densities. However, most white OLEDs that rely on co-doping red, green, and blue emitters within a single electroluminescent (EL) unit suffer from uncontrolled energy transfer, low device efficiency, poor emission balance [17], and limited spectral stability [18]. Tandem OLEDs formed by stacking multiple color-specific EL units can alleviate these issues and improve efficiency. Fung and his team have successfully developed a luminescent object composite strategy, successfully achieving the red, green, and blue color mixture of white light devices through the stacking of two units [19]. The tandem structure also helps to increase the lifespan of the device and reduce internal optical losses [20]. Blue emission plays a very important role in high-quality displays [21], but the limited lifetime of the blue sub-unit in RGB-stacked white OLEDs remains a fundamental bottleneck [22].

Replacing the unstable blue OLED with a long-lived GaN-based LED offers a compelling route to overcoming the intrinsic stability limitations of tandem white OLEDs. GaN-based LEDs provide a compelling solution. They exhibit excellent operational stability and high external quantum efficiency in the blue region, making them ideal candidates to replace the unstable blue unit in white OLEDs [23]. Current research has successfully achieved the alternating drive of two units through etching and vacuum deposition, while the structure is not suitable for integration onto current microdisplays [24]. However, integrating GaN-LEDs and vacuum-deposited OLEDs within a single tandem architecture has remained unexplored, primarily due to their incompatible fabrication processes: GaN-LEDs require high-temperature epitaxy, whereas OLEDs demand low-temperature organic deposition. In addition, significant current mismatch between inorganic and organic sub-units further complicates device integration.

In this work, we present the first hybrid tandem white light-emitting diode (HT-WLED), in which a GaN-based blue EL unit and an organic EL unit co-doped with red and green phosphorescent dopants are electrically connected through an interface-engineered charge-generation unit (CGU). The CGU consists of an ITO/HAT-CN/LiNH_2_-doped Bphen trilayer structure, where 1,4,5,8,9,11-hexaazatriphenylenehexacarbonitrile (HAT-CN) optimizes the interfacial energetics and morphology of indium tin oxide (ITO), and the LiNH_2_-doped bathophenanthroline (Bphen) layer provides efficient n-type charge generation for bridging the inorganic and organic sub-units. As verified by AFM and KPFM measurements, HAT-CN significantly reduces the interfacial roughness and deepens the surface potential, thereby promoting more efficient charge generation and balanced carrier injection. Benefiting from these synergistic improvements, the hybrid tandem device achieves near-ideal voltage summation, a twofold increase in current efficiency, and markedly reduced spectral drift over a wide current range. Furthermore, the same strategy proves effective when applied to a blue–yellow complementary-emission system, demonstrating the versatility and generality of the interface-engineered hybrid GaN–OLED architecture.

## 2. Experiments and Characterizations

### 2.1. Device Fabrication 

*GaN-LED*: The hybrid LED consists of GaN-LEDs and OLEDs connected in a tandem structure with a charge generation layer. The GaN-LEDs were prepared by the epitaxial growth technology using chemical vapor deposition on the patterned sapphire substrates (PSS), with a structure of μ-GaN (3.0 μm), highly Si-doped n-GaN (2 μm), lowly Si-doped n-GaN (250 nm), InGaN/GaN transition layer (250 nm), InGaN/GaN MQW (250 nm) for emission at 456 nm, p-AlGaN (60 nm), and p-GaN (200 nm). After the deposition, the mesa was etched downward until high Si-doped n-GaN was exposed, and an ITO layer of 150 nm was deposited. Then, a 400 nm thick SiO_2_ isolation layer was coated by electron-beam deposition, covering all the areas. The MQW and p-GaN parts were fabricated by etching the SiO_2_ with a small area of 3.5 × 4.5 mm^2^. Additionally, an Au layer (1.7 μm) for the n-contact pad was deposited onto a small window by also etching the SiO_2_ isolation layer. At last, the substrate was ground and polished to a thickness of 400 μm.

*OLED*: The OLEDs were fabricated by vacuum deposition technology. The organic and inorganic materials were all from commercial companies and without further purification. All the functional layers were fabricated on pretreated indium tin oxide (ITO) substrates. The glass substrates coated with ITO layers were sequentially cleaned ultrasonically with acetone and ethanol and then dried in an oven at 110 °C for 1 h. After being treated with ultraviolet-ozone plasma, the substrates were transferred into the evaporation chamber. The OLEDs were fabricated by vacuum deposition under a pressure of 4 × 10^−6^ Torr. The deposition rates and doping concentrations of the organic functional materials and metal electrodes were controlled and monitored in situ using a film thickness calibration instrument. The co-evaporation doping ratio for the emissive material Ir(MDQ)_2_(acac) and Ir(ppy)_2_(acac) were 1 vol% and 7 vol%, respectively. The LiNH_2_ (10 vol%)/Bphen layer was fabricated by thermal co-evaporation under a high level of vacuum (<5 × 10^−6^ Torr). Deposition rates were precisely controlled using an independently calibrated quartz crystal oscillator with ±0.01 Å/s accuracy. The Al electrode was deposited onto the organic functional layers at a rate of 6 Å/s with a thickness of 100 nm. After the deposition of all organic and metal electrode layers, four emitting pixels with an emission area of 10 mm^2^ each were formed on one substrate.

*HT-WLED*: The HT-WLEDs were fabricated by connecting the GaN-LED and OLED. All the functional layers were fabricated, which covered all of the ITO area of the GaN-LED in the evaporation chamber. All the deposition conditions were the same as the OLEDs. The deposition rates of organic materials were all 1 Å/s, except the emissive material Ir(MDQ)_2_(acac) with Ir(ppy)_2_(acac) with 0.1 Å/s, 0.6 Å/s, respectively, and PO-01 with 0.7 Å/s. The Al electrode was deposited onto the organic functional layers at a rate of 6 Å/s with a thickness of 100 nm, connecting the Au anode. The emitting area of the OLED was controlled to be the same as that of the GaN-LED by adjusting the overlapping area of the metal and organic layers.

### 2.2. Characterizations

*EL performance*: After the deposition of functional layers, hybrid device characterization was carried out at room temperature. The current density, driving voltage, and luminance of the devices were measured using a testing system consisting of a Keithley 2400 source meter (Keithley, Solon, OH, USA, Keithley 2400) and a spectroradiometer (Photo Research, New York, NY, USA, PR 655). The electroluminescence (EL) spectra were measured using a spectral scan photometer PR 655.

*AFM*: Surface profiles were measured by AFM (Bruker, Billerica, MA, USA, Veeco Dimension icon) in a tapping mode with scanning areas of 2.0 µm × 2.0 µm.

*KPFM*: KPFM measurement was collected by using AFM (Asylum Research, Goleta, CA, USA, Cypher S) to investigate the surface potentials.

*SEM*: Using a glass cutter, the prepared HT-WLED was cut, and then it was split apart by hand. A scanning electron microscope (SEM) was used to investigate the cross-section image with an accelerating voltage of 8 kV (Hitachi, Tokyo, Japan, SU8230).

## 3. Results and Discussion

### 3.1. EL Performances of Single LEDs

**Figure 1:**
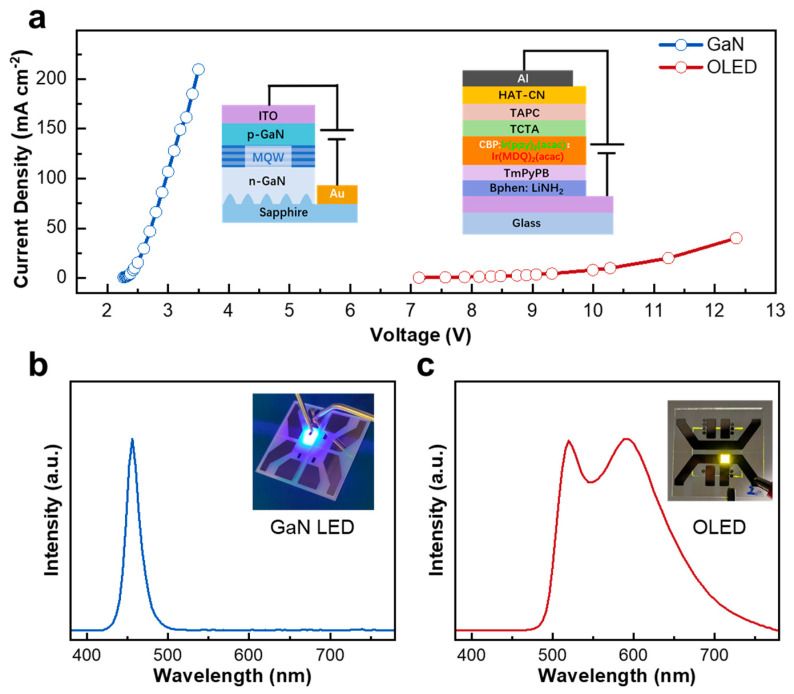
(**a**) Current density–voltage (J–V) curves of the blue GaN-LED and the red–green OLED sub-units. Normalized electroluminescence (EL) spectra of the GaN-LED (**b**) and the R/G-mixed OLED (**c**). Inset: photographic images of the blue GaN-LED and the yellowish OLED.

To evaluate the feasibility of constructing an HT-WLED, we first characterized the EL performance of the individual GaN and OLED sub-units. The GaN-based blue LED (inset in Figure 1a) consists of a conventional n–i–p structure grown on sapphire by metal–organic chemical vapor deposition, comprising an n-GaN layer, multiple InGaN/GaN quantum wells, and a p-GaN layer, followed by mesa formation and metallization through conventional photolithographic processing (see Figure S1 in the supporting information). The red–green OLED (inset in Figure 1a) was fabricated on ITO substrates through thermal evaporation and incorporated a co-doped emissive layer based on bis(2-phenylpyridine)(acetylacetonato)iridium(III)(Ir(ppy)_2_(acac)) and bis(2-methyldibenzo-[f,h]quinoxaline)(acetylacetonate)iridium(III)(Ir(MDQ)_2_(acac)) phosphorescent dopants. The molecular structures of all organic compounds used in the OLED are provided in Figure S2.

Figure 1a shows the J–V characteristics of the two single-unit devices. The GaN-LED exhibits a low turn-on voltage and reaches a current density of 200 mA cm^−2^ at approximately 3.5 V. In contrast, the OLED sub-unit delivers only ~50 mA cm^−2^ under 12 V. This large difference in electrical characteristics highlights the inherent current mismatch between the two sub-units. Furthermore, the radiance of GaN-LEDs and OLEDs varies differently with changes in the current density, which will affect the matching of spectral intensity (Figure S3). This must be carefully addressed to enable efficient tandem operation.

However, the blue emission from GaN-LED and the red–green emission from OLED have a complementary nature for spectral combination. The corresponding EL spectra are presented in Figure 1b. The GaN-LED shows a narrow blue emission centered at 456 nm with a full width at half-maximum of 35 nm. The OLED exhibits two peaks at 532 nm and 618 nm, arising from Ir(ppy)_2_(acac) and Ir(MDQ)_2_(acac), respectively, yielding a yellowish overall emission (Figure 1c). The complementary nature of the blue and red–green emissions provides a promising basis for achieving high-quality white emission in a tandem configuration.

### 3.2. EL Performances of Hybrid Tandem White LEDs

**Figure 2:**
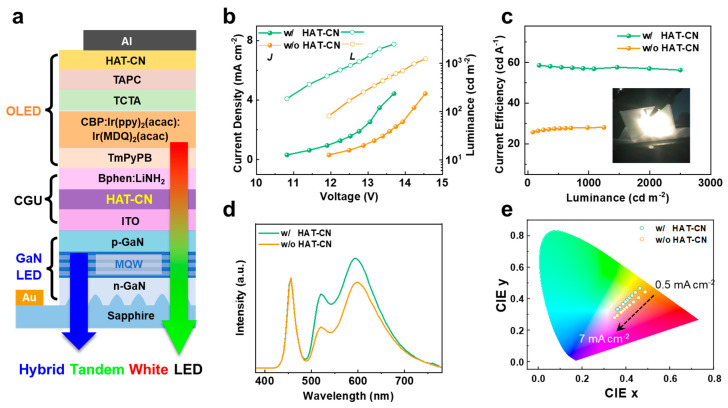
(**a**) Schematic device architecture of the hybrid tandem LED. (**b**) J–V–L characteristics, (**c**) current-efficiency versus luminance, (**d**) EL spectra at a current density of 2 mA cm^−2^, (**e**) CIE coordinate evolution as a function of current density of the hybrid tandem LEDs with and without HAT-CN incorporated in the CGU.

Based on the distinct electrical characteristics and spectral outputs of the GaN and OLED sub-units described above, we next constructed HT-WLEDs by vertically stacking the two EL units (Figure 2a). To establish efficient electrical coupling between the inorganic and organic units, a charge-generation unit (CGU) was introduced at their interface. CGU is a connection structure in tandem devices, which connects different units and generates carriers under voltage to enable emission simultaneously [25,26]. We systematically examined the influence of incorporating HAT-CN into the CGU by comparing devices fabricated with and without this interfacial layer.

Figure 2b presents the current density–voltage (J–V) characteristics of the hybrid tandem devices. The control device without HAT-CN exhibits a noticeably higher operating voltage, reflecting an inefficient charge transfer at the unmodified interface and deviating significantly from the ideal voltage summation. In contrast, the device incorporating HAT-CN shows a lower turn-on voltage and higher current density across the entire bias range, indicating a more efficient charge generation within the CGU. Importantly, near-ideal voltage addition is achieved: the turn-on voltage of the tandem closely matches the sum of the individual GaN and OLED voltages, confirming a proper electrical series connection. From the energy level diagram, all layers in the tandem device were arranged in an ideal energy level sequence to regulate the carrier transport within the device (Figure S4), further proving the excellent charge generation capability of the modified CGU.

The corresponding luminance–voltage (L–V) trends in Figure 2c further highlight the impact of inserting HAT-CN. Under the same driving voltage, the HAT-CN device achieves substantially higher luminance—more than twice that of the control device at typical operating conditions. Consequently, the current efficiency of the hybrid tandem LED increases from 28.1 cd A^−1^ to 58.6 cd A^−1^ upon incorporating HAT-CN, corresponding to nearly a twofold improvement. The external quantum efficiency (EQE) of the tandem device was also improved (Figure S5). These trends collectively demonstrate that HAT-CN reduces the interfacial barrier within the CGU, enhances charge-generation efficiency, and enables balanced carrier injection into both sub-units—providing an electrical basis for improved emission behavior.

The evolution of the emission spectra with increasing current density is shown in Figure 2e and Figure S6. At low current densities, both devices exhibit OLED-dominated orange emission because the GaN-LED contributes minimally at weak driving currents. As the current increases, the contribution from the GaN blue emission becomes progressively stronger, shifting the overall output toward the white-light region. Compared with the control device, the HAT-CN device shows much smaller changes in the relative intensities of the blue and red–green components, leading to a significantly reduced shift in its CIE coordinates. These results indicate that the CGU interface strongly influences the distribution of carriers between the two EL units and thereby regulates their relative emission contributions across the operating range. In particular, the reduced CIE shift in the HAT-CN device reflects the improved charge balance enabled by the HAT-CN–modified CGU, demonstrating that interfacial energetic and morphological optimization directly contributes to more controlled and predictable emission behavior in the hybrid tandem structure.

In addition to steady-state characteristics, we also evaluated the operational stability of the tandem devices under a constant current of 0.2 mA. As shown in Figure S7, the device incorporating HAT-CN exhibits a T_70_ lifetime of 69 min, more than twice that of the control device (33 min). This extended operational lifetime indicates that the improved carrier balance enabled by the HAT-CN-modified CGU not only enhances instantaneous emission efficiency but also mitigates degradation pathways associated with injection imbalance and localized exciton accumulation. These results further underscore the importance of interfacial engineering within the CGU in determining both the efficiency and stability of hybrid GaN–OLED tandem operation.

### 3.3. Interfacial Mechanisms Underlying the Improved CGU Performance

**Figure 3:**
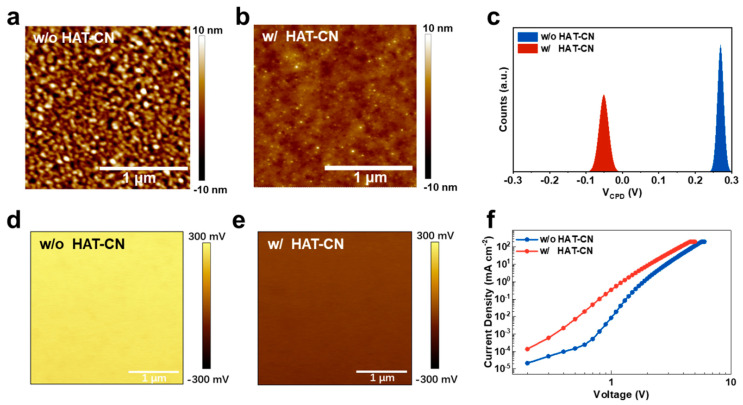
(**a**,**b**) AFM height images of ITO surfaces with and without HAT-CN, showing a substantial reduction in surface roughness from 2.69 nm to 0.73 nm after HAT-CN deposition. (**c**) Surface potential distributions for ITO on GaN substrate with and without HAT-CN, indicating a notable increase in work function (from 0.27 V to −0.05 V) for HAT-CN–modified ITO. Corresponding kelvin probe force microscopy (KPFM) maps for ITO on GaN substrate (**d**) with and (**e**) without HAT-CN. (**f**) Electron-only devices with and without HAT-CN.

To uncover why HAT-CN so effectively improves tandem device performance, we first examined the interfacial morphology using atomic force microscopy (AFM). As shown in Figure 3a,b, the deposition of HAT-CN significantly reduces the root-mean-square roughness from 2.69 nm to 0.73 nm. A smoother interface suppresses local field concentration and reduces trap-assisted recombination, enabling more uniform charge generation. The cross-sectional SEM image shows smooth and well-defined interfaces without observable interlayer diffusion (Figure S8), confirming excellent film uniformity.

Kelvin probe force microscopy (KPFM) measurements (Figure 3d,e) provide complementary insight into the interfacial energetics. The surface potential shifts from 0.27 V for bare ITO to –0.05 V after HAT-CN deposition, corresponding to an increase in the effective work function [27]. In addition to the shift in the mean potential, the potential fluctuation amplitude is reduced, suggesting a more homogeneous energetic landscape. This deeper and more uniform work function strengthens the built-in electric field within the CGU, thereby facilitating electron extraction from GaN and hole injection into the OLED sub-unit.

To quantify the built-in potential within the CGU, we performed capacitance–voltage (C–V) measurements on a standalone CGU structure: ITO/HAT-CN (60 nm)/LiNH2-Bphen (60 nm, 10 vol%)/Al (100 nm). From the linear region of the 1/C^2^-V plot (Figure S9), we extract a built-in voltage of ~0.69 V. This value is consistent with the 0.32 V surface-potential difference obtained via KPFM, considering measurement geometry differences. Although the exact depletion width cannot be experimentally determined under current constraints, assuming a typical interfacial depletion width of ~2 nm, we estimate an internal field strength of 1.6–3.5 MV cm^–1^. This MV-level enhancement strongly promotes hole extraction from GaN and electron injection into the OLED, explaining the near-ideal voltage summation in the tandem device.

To further probe the injection characteristics of the CGU, we fabricated electron-only devices (EODs) with the structure ITO/with or without HAT-CN/LiNH_2_–doped/BPhen/TmPyPB/LiNH_2_–doped Bphen/Al. As shown in Figure 3c, the HAT-CN-containing device follows the typical space-charge-limited current (SCLC) behavior over the measured voltage range, indicating efficient charge injection and minimal interfacial barriers. In contrast, the device without HAT-CN exhibits a trap-filled limited (TFL) current region, showing pronounced deviation from SCLC transport. This behavior demonstrates the presence of interfacial traps and a higher injection barrier in the absence of HAT-CN. The transition from TFL to SCLC behavior upon inserting HAT-CN confirms that the interfacial layer effectively lowers the injection barrier and enables near-Ohmic contact at the CGU. To directly assess the charge-generation capability of the CGU, standalone CGU devices were fabricated (Figure S10). The HAT-CN–containing structure exhibits markedly enhanced current density at lower voltages, consistent with the efficient charge generation observed in the tandem devices. Taken together, these observations show that HAT-CN enables a more balanced carrier generation between the GaN and OLED sub-units, providing a mechanistic explanation for the improved voltage summation, higher luminance, enhanced current efficiency, and reduced spectral variation, observed in Figure 2.

### 3.4. Other Hybrid Tandem Strategy: Blue–Yellow White LEDs

**Figure 4:**
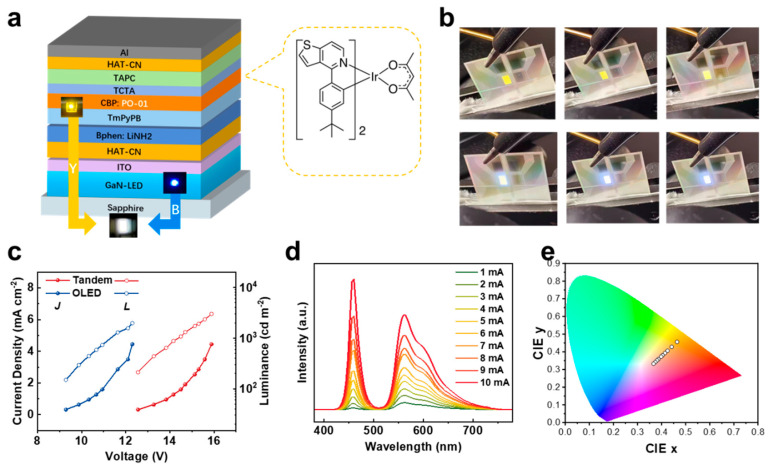
(**a**) Device structure of the GaN/PO-01 hybrid tandem LED employing the same CGU design. (**b**) Photographs of device emission under increasing current. (**c**) J–V–L curves of the GaN/PO-01 tandem LED and PO-01-based single OLED. (**d**) EL spectra and (**e**) CIE coordinates evolution of the GaN/PO-01 hybrid tandem LED as a function of current density.

To further verify the generality of the proposed hybrid tandem strategy, we fabricated a blue–yellow tandem device using bis(4-phenylthieno[3,2-c]pyridinato-N,C2′) (acetylacetonate)iridium(III) (PO-01) as the yellow emitter in the organic EL unit (Figure 4a). As shown in Figure 4b–c, the device exhibits nearly ideal voltage addition, with a ΔV of 3.27 V—closely matching the operating voltage of the single GaN-LED—indicating efficient charge generation at the CGU. At a current density of 4.44 mA cm^–2^, the yellow OLED and the hybrid tandem LED deliver luminance values of 1967 cd m^–2^ and 2983 cd m^–2^, respectively.

The corresponding EL spectra (Figure 4d) display complementary 456 nm blue and 560 nm yellow emission, producing visually high-quality white light at elevated currents (Figure 4e). Correspondingly, the CIE coordinates shift from a warm-white region at low current densities toward neutral-white as the contribution from the GaN sub-unit increases (Figure 4e). At 10 mA cm^–2^, the device reaches CIE coordinates of (0.36, 0.33), illustrating tunable white emission across the operating range.

This tandem device also shows improved efficiency compared with the single yellow OLED (Figure S11), consistent with the same enhancement observed in the red–green system. The consistent improvements in voltage addition, luminance, spectral composition, and efficiency confirm that the HAT-CN–based CGU is broadly applicable across different OLED architectures and emission combinations.

### 3.5. Advantages and Limitations of CGU and HT-LED

We have demonstrated the first successful electrical integration of a GaN-based LED and a vacuum-deposited OLED within a single tandem structure. By designing a CGU composed of ITO/HAT-CN/LiNH_2_-doped Bphen, the interfacial morphology and built-in electric field are optimized, enabling efficient charge generation and balanced injection. Consequently, the hybrid tandem device achieves near-ideal voltage addition and an approximate twofold increase in current efficiency (from 28.1 to 58.6 cd A^−1^), validating the effectiveness of this strategy in enhancing device efficiency and spectral stability. The efficiency distribution of the three devices (Figure S12) also indicates the good repeatability of our hybrid tandem strategy. Although the preparation process of this tandem structure is rather complex compared to the dual-emission white OLED [28] that has gradually attracted the attention of researchers at present. However, compared to the current efficiency of approximately less than 10 cd A^−1^, the HT-LEDs we have currently prepared still have a relatively high level of current efficiency, and do not require complex molecular design and synthesis. They are compatible with commercial OLED luminescent materials and still have advantages.

The proposed CGU design also exhibits good generality, proving equally applicable to OLED units employing the yellow phosphorescent emitter PO-01, successfully enabling a spectrally tunable blue–yellow complementary white device. This lays a solid foundation for future development of compact white-light microdisplays. However, limitations remain. The lifetime of the OLED unit and CGU still lags behind that of the GaN-LED, limiting overall device longevity. Furthermore, the high operating voltage of the OLED section and its lower radiance compared to the GaN-LED at high current densities are issues that require further investigation and resolution.

## 4. Conclusions

This work demonstrates that targeted interfacial engineering enables efficient electrical coupling between GaN and organic EL units in a hybrid stacked architecture. The HAT-CN–based charge-generation interface simultaneously improves energetic alignment and interfacial uniformity, resulting in balanced carrier generation and more controlled recombination in the OLED sub-unit. These improvements suppress spectral drift and stabilize white emission, underscoring the central role of interface design in hybrid tandem devices. The same strategy also applies to blue–yellow complementary emission, confirming its generality across different OLED configurations. Overall, this approach establishes a clear and practical design principle for integrating dissimilar inorganic and organic emitters into compact and electrically efficient hybrid white-light sources.

## Data Availability

The original contributions presented in this study are included in the article/Supplementary Material. Further inquiries can be directed to the corresponding authors.

## Supplementary Materials

The following supporting information can be downloaded at: https://www.mdpi.com/article/10.3390/ma18245684/s1, Figure S1: Fabrication flow of the GaN-LED; Figure S2: Molecular structures of all organic compound used in the OLEDs; Figure S3: Current density versus radiance characteristics of GaN-LED and OLED; Figure S4: Energy-level diagram for the whole tandem device; Figure S5: External quantum efficiency of (a) HT-LED with and without HAT-CN, (b) HT-LED based on GaN and PO-01; Figure S6: (a) Normalized EL spectra and (b) EL spectra of the tandem WLED without HAT-CN in the CGU. (c) Normalized EL spectra and (d) EL spectra of the tandem WLED with HAT-CN in the CGU; Figure S7: Luminance decay of the tandem WLED (without sealing) as a function of aging time; Figure S8: Cross-section scanning electron microscopy (SEM) image of the tandem device; Figure S9: 1/C^2^–V characteristics of CGU-only device; Figure S10: Current density versus voltage characteristics of CGU-only device; Figure S11: CE versus luminance characteristics of HT-LED based on GaN and PO-01. Figure S12: CE versus luminance characteristics of HT-LED based on GaN and PO-01. References [29,30,31,32] are cited in the supplementary materials.
