# Supplementary material for: Hybrid Tandem White Light-Emitting Diodes Based on GaN and Organic Emitters
Source: Materials (Basel). 2025 Dec 18;18(24):5684. doi: 10.3390/ma18245684 (PMC12734585; doi:10.3390/ma18245684)
Supplement: Supplementary file 1 [file materials-18-05684-s001.zip › materials-4042851-supplementary.pdf]

Hybrid Tandem White Light-Emitting Diodes Based on GaN and Organic Emitters

Jin-Zhe Xu[1], Xiao-Zhao Zhu[2], Feng Zhai[3], Wei-Zhi Liu[1], Dong-Ying Zhou[1,], Liang-Sheng Liao[1,4,*]*

[1] Institute of Functional Nano & Soft Materials (FUNSOM), Jiangsu Key Laboratory for Carbon-Based Functional Materials & Devices, Soochow University, Suzhou, Jiangsu 215123, China

[2] Institute of Organic Optoelectronics (IOO), Jiangsu Industrial Technology Research Institute (JITRI), Suzhou, Jiangsu 215215, China

[3] Key Laboratory of Optoelectronic Technology and Systems (Ministry of Education), College of Optoelectronic Engineering, Chongqing University, Chongqing 400044, China

[4] Macao Institute of Materials Science and Engineering, Macau University of Science and Technology, Taipa, Macau SAR 999078, China

* E-mail: dyzhou@suda.edu.cn; lsliao@suda.edu.cn

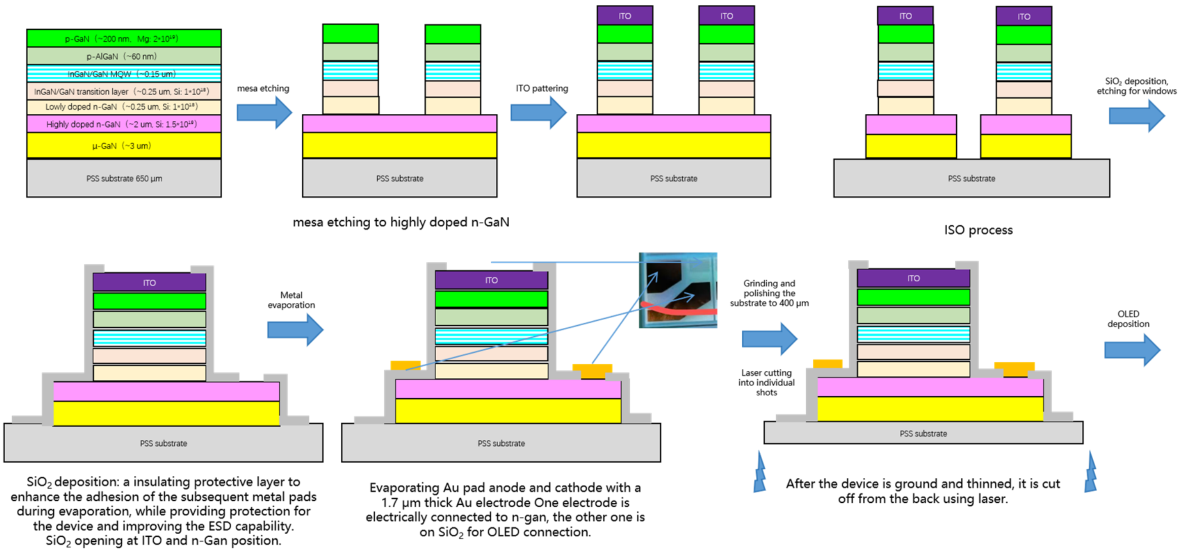

Figure S1. Fabrication flow of the GaN LED.

µ-GaN: The ~3.0 µm µ-GaN buffer layer is critical for reducing threading dislocation densities. Its thickness represents a standard optimization, reducing epitaxial stress between the substrate and n-GaN.

n-GaN: The ~2.25 µm n-GaN layers is essential for lateral current spreading and supplying electrons to the active region. Its thickness is optimized to minimize series resistance. The highly doped n-GaN layer drastically increases the free-electron concentration, resulting in very high electrical conductivity. The lowly doped n-GaN upper layer (~250 nm) reduces electron overflow, protecting the active region and enhancing overall efficiency.

MQW: The InGaN/GaN MQW layers are kept extremely thin (each well ~2–3 nm) to exploit strong quantum confinement, which tightly concentrates electron and hole wavefunctions, thereby maximizing radiative recombination efficiency and precisely tuning the emission to ~456 nm.

p-AlGaN: This layer (~60 nm) is designed with higher bandgap and polarization to create an energy barrier that prevents electron overflow from the MQW, thereby confining carriers within the active region for efficient recombination. If the p-AlGaN layer is excessively thick, it will introduce higher working voltage.

p-GaN: Its thickness (~20 nm) is optimized to balance low series resistance for hole injection with minimal optical absorption. It also provides a suitable surface for the subsequent ITO current-spreading electrode.

Electrodes: The thickness of ITO (150 nm) is optimized to balance low sheet resistance for uniform current spreading with high transparency, enabling both efficient hole injection and minimal optical loss from the GaN sub unit. Regarding the Au pad (1.7 µm thickness), its substantial thickness ensures robust, low-resistance ohmic contact and provides physical durability against probes during

testing and protects the underlying semiconductor layers from damage.

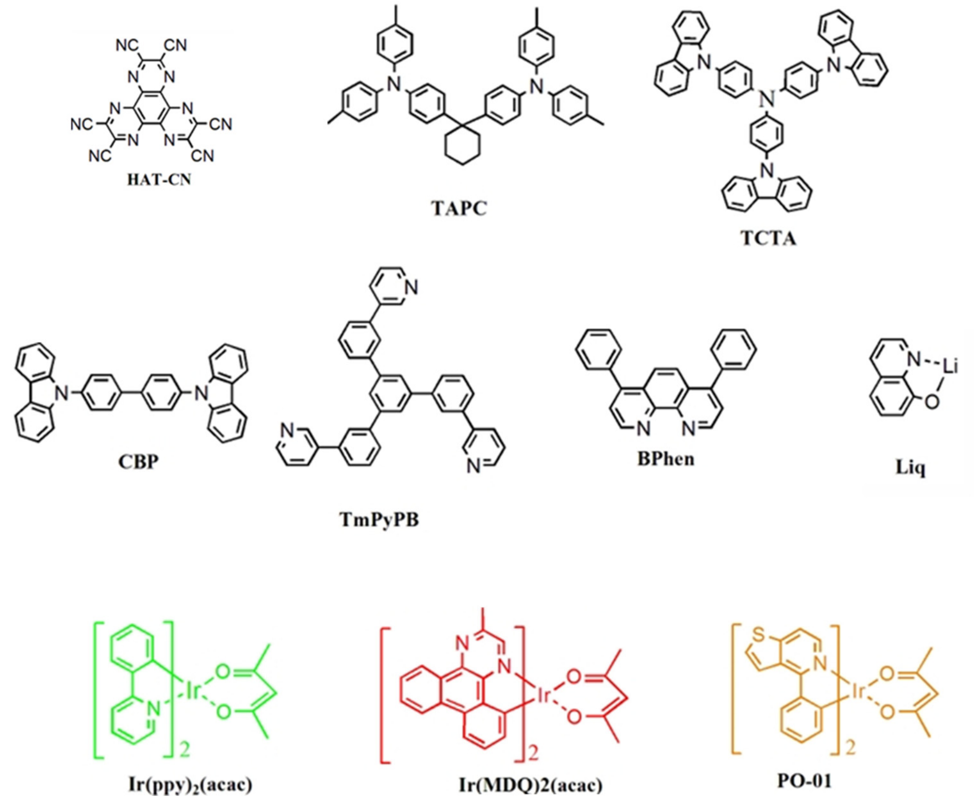

Figure S2. Molecular structures of all organic compound used in the OLEDs.

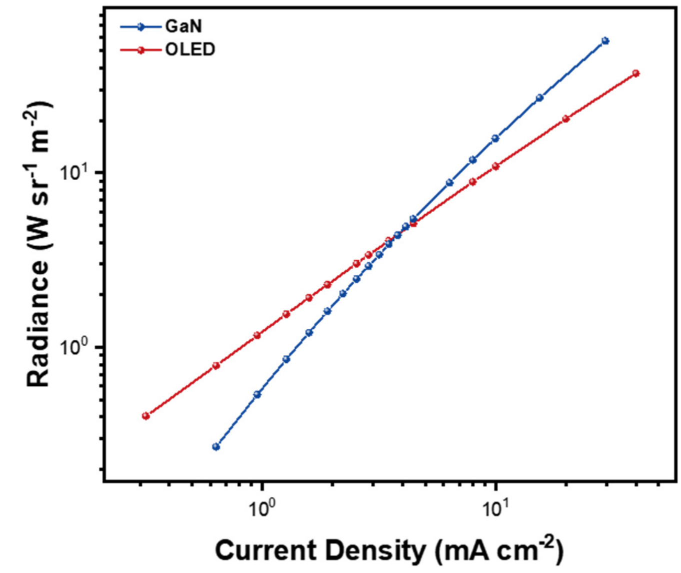

Figure S3. Current Density versus radiance characteristics of GaN-LED and OLED.

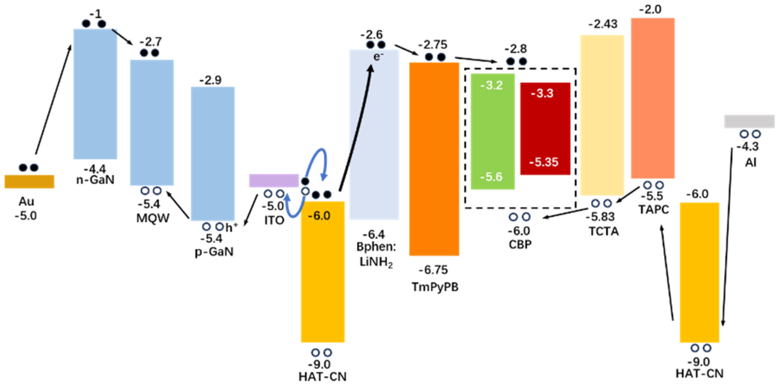

Figure S4 Energy-level diagram for the whole tandem device.

The diagram integrates the relevant energy levels from the literature for all material components involved [S1-S4]

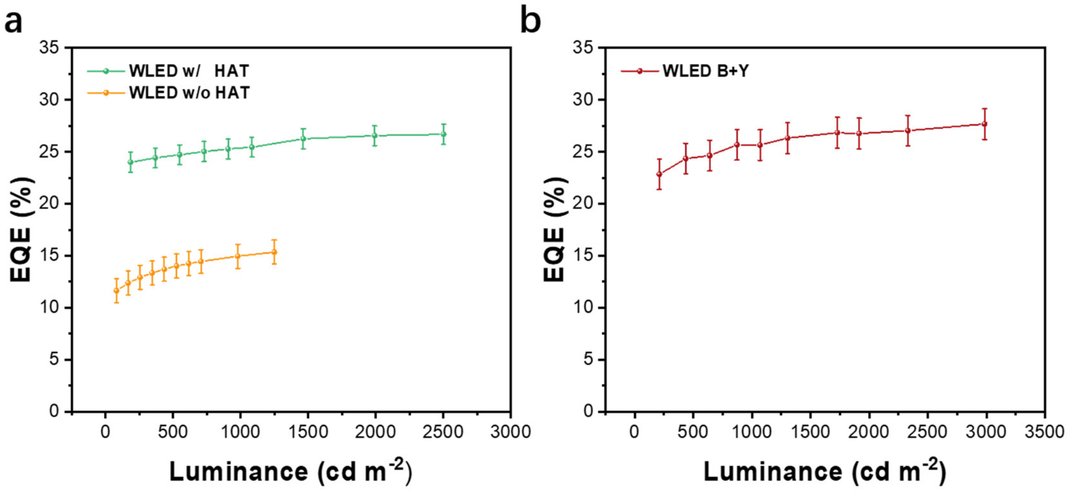

Figure S5 External quantum efficiency of (a) HT-LED with and without HAT-CN, (b) HT-LED based on GaN and PO-01.

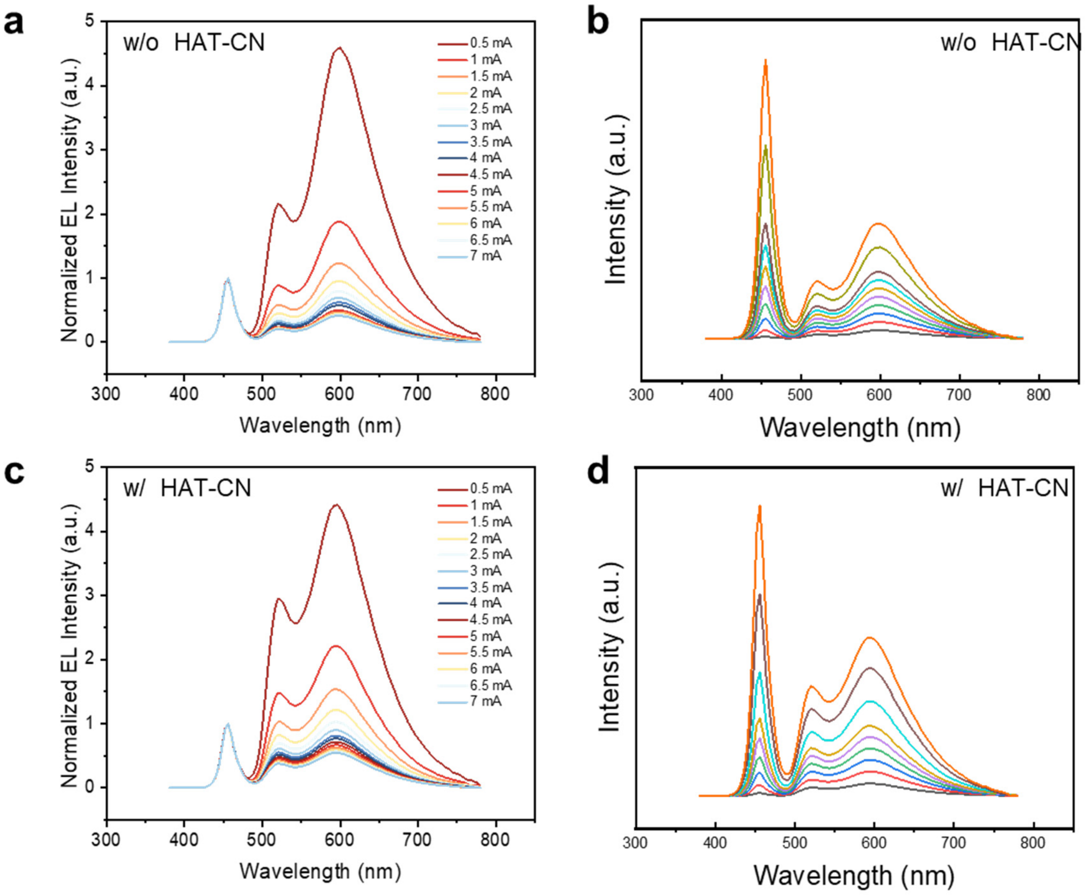

Figure S6. (a) Normalized EL spectra and (b) EL spectra of the tandem WLED without HAT-CN in the CGU. (c) Normalized EL spectra and (d) EL spectra of the tandem WLED with HAT-CN in the CGU.

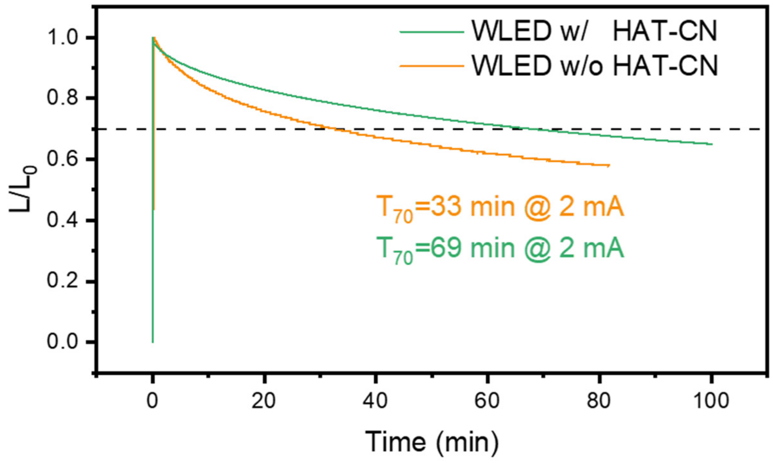

Figure S7. Luminance decay of the tandem WLED (without sealing) as a function of aging time.

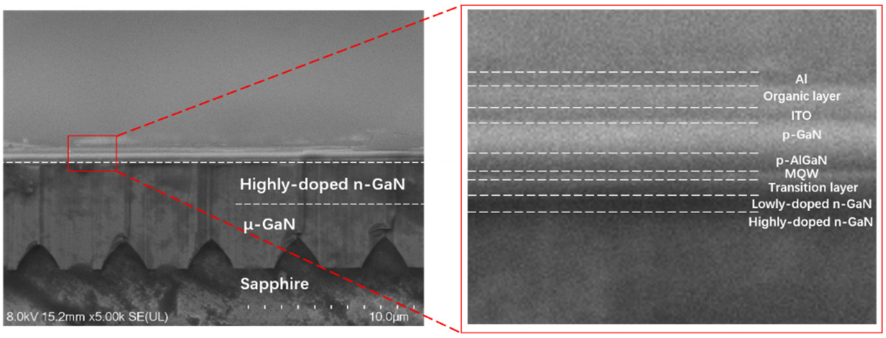

Figure S8. Cross-section scanning electron microscopy (SEM) image of the tandem device.

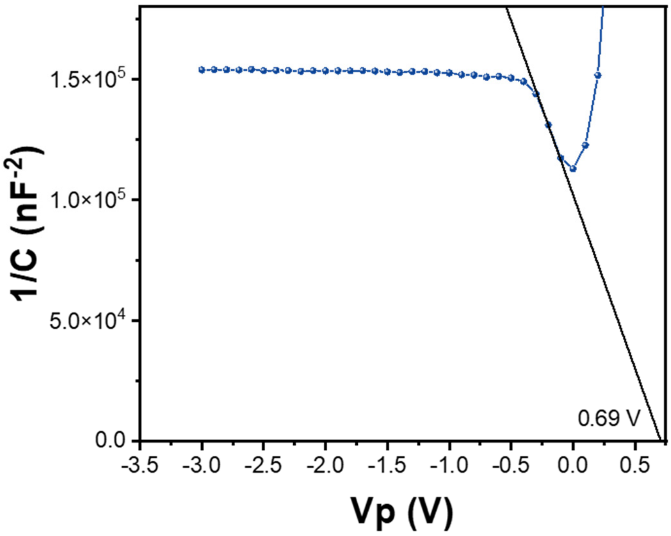

Figure S9. 1/C_2 - V characteristics of CGU-only device.

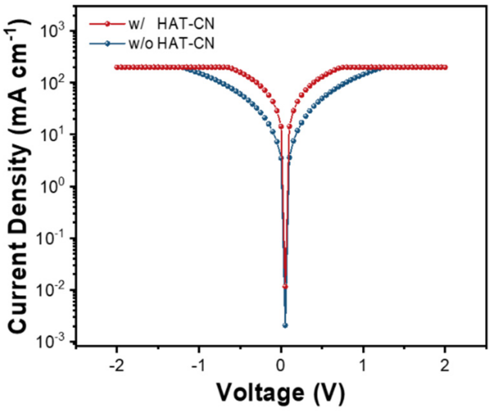

Figure S10. Current density versus voltage characteristics of CGU-only device.

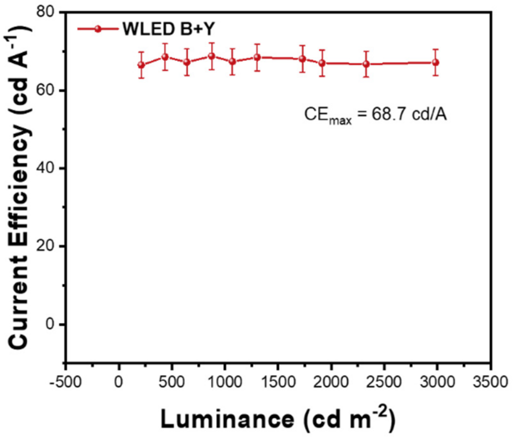

Figure S11. CE versus luminance characteristics of HT-LED based on GaN and PO-01.

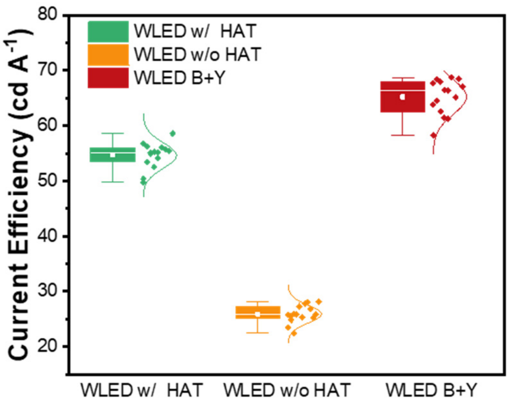

Figure S12. The current efficiency distribution of devices for three tandem devices.

Reference

[S1] Ding, L.; Sun, Y.-Q.; Chen, H.; Zu, F.-S.; Wang, Z.-K.; Liao, L.-S. A novel intermediate connector with improved charge generation and separation for large-area tandem white organic lighting devices. *J. Mater. Chem. C* **2014**, *2*, 10403–10408.

[S2] Chang, Y.-L.; Wang, Z. B.; Helander, M. G.; Qiu, J.; Puzzo, D. P.; Lu, Z. H. Enhancing the efficiency of simplified red phosphorescent organic light emitting diodes by exciton harvesting. *Org. Electron.* **2012**, *13*, 925–931.

[S3] Wu, S.; Li, S.; Sun, Q.; Huang, C.; Fung, M.-K. Highly Efficient White Organic Light-Emitting Diodes with Ultrathin Emissive Layers and a Spacer-Free Structure. *Sci. Rep.* **2016**, *6*, 25821.

[S4] Kim, B. S.; Lee, J. Y. Engineering of Mixed Host for High External Quantum Efficiency above 25% in Green Thermally Activated Delayed Fluorescence Device. *Adv. Funct. Mater.* **2014**, *24*, 3970–3977.
